# Newly Designed Hydrolysis Acidification Flat-Sheet Ceramic Membrane Bioreactor for Treating High-Strength Dyeing Wastewater

**DOI:** 10.3390/ijerph16050777

**Published:** 2019-03-04

**Authors:** Yue Jin, Dunqiu Wang, Wenjie Zhang

**Affiliations:** 1 College of Civil Engineering and Architecture, Guilin University of Technology, Guilin 541004, China; 2011016@glut.edu.cn; 2 Guangxi Key Laboratory of Environmental Pollution Control Theory and Technology, Guilin University of Technology, Guilin 541004, China

**Keywords:** hydrolysis acidification, MBR, membrane fouling, decolorization, microbial diversity, seed sludge

## Abstract

Cost-effective treatment of dyeing wastewater remains a challenge. In this study, a newly designed hydrolysis acidification flat-sheet ceramic membrane bioreactor (HA-CMBR) was used in treating high-strength dyeing wastewater. The start-up phase of the HA-CMBR was accomplished in 29 days by using cultivated seed sludge. Chemical oxygen demand (COD) removal rate reached about 62% with influent COD of 7800 mg/L and an organic loading rate of 7.80 kg-COD/(m^3^·d). Chromaticity removal exceeded 99%. The results show that the HA-CMBR has good removal performance in treating dyeing wastewater. The HA-CMBR could run with low energy consumption at trans-membrane pressure (TMP) <10 kPa due to the good water permeability of the flat-sheet ceramic membrane. New strains with 92%–96% similarity to *Alkalibaculum bacchi*, *Pseudomonas* sp., *Desulfovibrio* sp., and *Halothiobacillaceae* were identified in the HA-CMBR. Microbial population analysis indicated that *Desulfovibrio* sp., *Deltaproteobacteria*, *Halothiobacillaceae, Alkalibaculum* sp., *Pseudomonas* sp., *Desulfomicrobium* sp., and *Chlorobaculum* sp. dominated in the HA-CMBR.

## 1. Introduction

China is the world’s largest producer of dyes, at more than 1.15 × 10^9^ kg annually [1]. Dye exports have brought some economic growth for China, but the dyeing production process generates large volumes of dyeing wastewater. The raw materials of dyeing production include benzene, naphthalene, and polycyclic aromatic hydrocarbons, but also contain heavy metals, salts, and other substances. Therefore, dyeing wastewater is characterized by complex compositions, deep color, and toxicity for living systems [2]. The discharge of untreated dyeing wastewater causes serious pollution to surrounding environments.

The cost-efficient treatment of dyeing wastewater has attracted much research interest [3,4,5]. At present, physical and chemical methods are usually used to treat dyeing wastewater [6]. However, such methods have high operating costs. In comparison, biological methods have the advantages of low running costs and being more environmentally friendly, and are expected to be widely used in treating dyeing wastewater [7]. Li et al. [8] reinforced an anaerobic hydrolysis–denitrification coupling process, and the coupling process showed efficient removal of nitrogen and aromatics. Hayat et al. [9] achieved 87% chemical oxygen demand (COD) removal efficiency in an anaerobic internal circulation (IC) reactor treating real textile industry wastewater. Manavi et al. [10] used aerobic granules to treat real dyeing wastewater and achieved 73% color removal and 68% COD removal with a cycle time of 24 h and an anaerobic-to-aerobic period of 3:1. Therefore, cultivated anaerobic microorganisms have been shown to be efficient for the degradation of dyeing wastewater. However, anaerobic microorganisms are easily lost from the reactor due to changing conditions such as linear velocity, granules breaking, gas floatation, etc., resulting in lower treatment performance. Membrane separation was deemed as an effective method [11]. Membrane bioreactor (MBR) is a wastewater treatment technology that can achieve high load and good removal efficiency through efficient separation of microorganisms [11,12]. Aerobic MBR (AeMBR) and anaerobic MBR (AnMBR) are the usual forms used in treating wastewater. AeMBR usually uses organic membranes as a membrane separation module, but for AnMBR, these can be damaged during prolonged operation [13]. Compared to conventional organic membranes, the advantages of ceramic membranes include high permeability performance, energy savings and cost reductions, long lifespan, good durability, and easy maintenance and management [14]. A flat ceramic sheet can be used as the recycling module of an AeMBR in the treatment of industrial and municipal wastewater [14,15,16]. Based on the advantages mentioned above and practical experience, flat-sheet ceramic membranes are expected to perform well in AnMBR. In addition, subsequent aerobic biological treatment is needed to meet the discharge standard in treating dyeing wastewater [6,7,8]. Among them, the biodegradability of wastewater after anaerobic treatment is one of the main factors in determining the removal efficiency. Anaerobic processes include hydrolysis acidification and methanogenesis. The hydrolysis acidification stage can effectively improve the biodegradability of dyeing wastewater in a shorter hydraulic retention time (HRT) [12]. Combined with aerobic biological treatment process, it is possible to meet the discharge standard of China [7,8]. Meanwhile, compared with anaerobic processes, the hydrolysis acidification process has the advantages of a simple structure and less investment. Thus, hydrolysis acidification based on flat-sheet ceramic membranes is suggested to be evaluated in treating dyeing wastewater. Nevertheless, there are no previous reports on hydrolysis acidification based on flat-sheet ceramic membranes used for treating dyeing wastewater.

The present study employs a flat ceramic sheet as the membrane module in a newly developed hydrolysis acidification flat-sheet ceramic membrane bioreactor (HA-CMBR) that was developed for treating dyeing wastewater. Anaerobic microorganisms were screened and seeded in the HA-CMBR. Treatment performance was evaluated using real dyeing wastewater, and 16S rRNA was employed to characterize changes in the microbial populations of the anaerobic sludge. The results might provide theoretical guidance and reference for engineering applications.

## 2. Materials and Methods

### 2.1. Wastewater Compositions

The wastewater sample used in this study was obtained from Weifang Ruicheng company (Shandong, China) that produces disperse, reactive, and acid dyes. The compositions of the dyeing wastewater are shown in Table 1. Due to the lack of nitrogen and phosphorus nutrients in the dyeing wastewater, NH_4_HCO_3_ and KH_2_PO_4_ are added as nitrogen and phosphorus nutrients in the required proportion.

### 2.2. Reactors

A schematic diagram of an HA-CMBR system is described by Zhang et al. [12]. Anaerobic microorganisms from a running hydrolysis acidification tank [17] were seeded in the main reactor. The permeate was discharged into the effluent tank using a filtration pump. The feed flow rate of HA-CMBR was 16 L/d with an HRT of 12 h. The HA-CMBR consisted of an influent tank, main reactor, warm water control system, and effluent tank. The influent was adjusted for pH in the influent tank and pumped into the bottom of the main reactor. The temperature of the main reactor and membrane pool was controlled at 34 ± 1 °C. The HA-CMBR was seeded with 2.5 L of sludge that had a mixed liquor volatile suspended solids (MLVSS) content of 10,000 mg/L.

### 2.3. Measurement Methods

Filtered COD (1 µm) based on dichromate was measured using the closed reflux colorimetric method [18]. Biochemical oxygen demand over a five-day (BOD_5_) and chroma were measured according to the standard methods [19]. Total nitrogen (TN) was determined with the persulfate method [20] using the ultraviolet spectrophotometric screening method for quantification of TN as NO_3_–N (i.e., oxidization product of persulfate digestion). Total phosphorus (TP) was measured according to Yue et al. [21,22]. The pH was measured using a pH meter (9010; Jenco, San Diego, CA, USA), and dissolved oxygen (DO) was measured using a DO meter (6010; Jenco, San Diego, CA, USA). Trans-membrane pressure (TMP) was recorded by a digital pressure sensor (SHANG YI, Foshan, China). Extracellular polymeric substances (EPS) and soluble microbial products (SMP) were analyzed according to Ramesh et al. [23].

### 2.4. Reactor Start-Up and Operation Parameters

The HA-CMBR was fitted with a flat-sheet ceramic membrane of normal pore size 0.1 μm and effective area 0.05 m^2^. The hydraulic residence time was set to 8 h (according to the main reactor). The main reactor was seeded with 3 L sludge that had a mixed liquor volatile suspended solids (MLVSS) content of 8000 mg/L.

### 2.5. Microbial Diversity

Eight sludge samples were collected during the study: Two samples for seed sludge (No. 1 and 2); and six sludge samples, one (No. 3) collected at the end of the start-up period, and five (Nos. 4–8) collected during the organic loading rates (OLR) (kg-COD/m^3^/d) of 1.30–1.45, 2.5–2.84, 3.73–4.10, 5.83–6.18, and 7.70–7.84, respectively. The samples were analyzed according to Zhang et al. [12]. DNA was extracted using the E.Z.N.A. Soil DNA Kit (OMEGA Biotec. D5625-01, Norcross, GA, USA) according to the manufacturer’s instructions. Partial 16S rRNA gene amplicons were generated using TransGen AP221-02 (TransStart Fastpfu DNA Polymerase, Axygen, New York, NY, USA) and ABI GeneAmp^®^ 9700 (ABI, Carlsbad, CA, USA). Duplicate PCR products were pooled and purified using the AXYGEN gel extraction kit (Axygen, New York, NY, USA). Sequencing was performed using the 454 GS FLX+ instrument (Roche, Branford, FL, USA), and the sequencing method manual XLR70 kit. PCR products were sequenced by the Shanghai Shenggong Company (Shanghai, China).The sequenced gene was compared with the National Center for Biotechnology Information (NCBI) website, and the denaturing gradient gel electrophoresis (DGGE) imaging was analyzed using Quantity One software (Manufacture, City, State abbrev., Country). 

Raw 454-pyrosequencing data were analyzed using Mothur v.1.40.0 (Ann Arbor, MI, USA). Operational taxonomic units (OTU) with 97% confidence were used for the construction of OTU rank–abundance curves.

### 2.6. Data Analysis

Data analysis was carried out using Origin 2017 software (OriginLab, Northampton, MA, USA). To explore the correlation between microbial diversity and environmental factors, detrended correlation analysis was performed on the species.

## 3. Results and Discussion

### 3.1. Reactor Performance

Diluted dyeing wastewater with COD concentration of 1300–1349 mg/L was used for the start-up of the HA-CMBR. The OLR was set at 1.3–1.5 kg-COD/m^3^/d. During the first three days of start-up, COD removal efficiency was poor with an effluent COD concentration of about 900 mg/L, after which the effluent COD gradually decreased during the following 22 days. A COD removal rate of 65% was achieved on day 23. From day 23, the COD removal rate remained stable in the following six days. Thus, the startup of HA-CMBR was deemed to be accomplished in 29 days. During the start-up period, a good chroma removal rate of 99% was achieved, which indicates that the microorganisms in the seed sludge are competent in decolorizing the dyeing wastewater.

After the start-up period, the effects of OLRs on the reactor were investigated. A total of six OLRs were set during the study. Influent COD concentrations and total removal rates were 1300–1450 mg/L and 57.5%–80.8%, respectively, in stage I; 2590–2840 mg/L and 58.7%–73.8% in stage II; 3730–4100 mg/L and 57.4%–69.1% in stage III; 5830–6180 mg/L and 55.7%–68.1% in stage IV; 7700–7840 mg/L and 60.2%–63.3% in stage V; and 9080–9110 mg/L and 36.3%–37.4% in stage VI.

**Figure 1:**
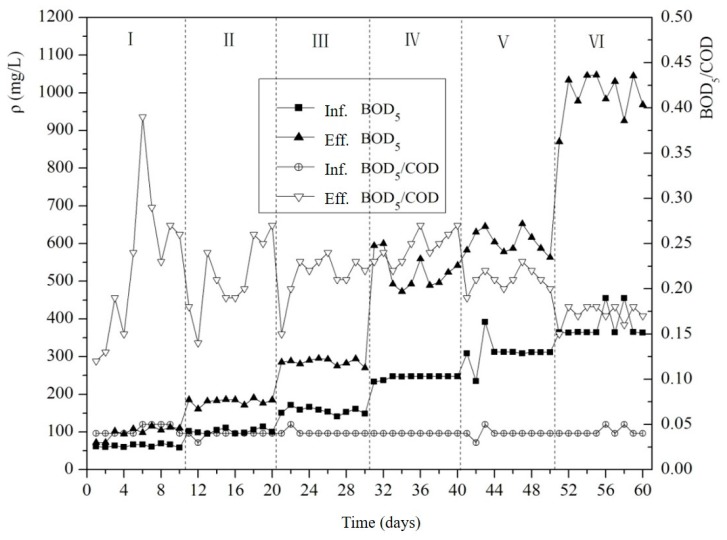
BOD_5_/chemical oxygen demand (COD) changes during the study (Inf., Influent; Eff., Effluent).

After treatment, the average BOD_5_/COD of stages I–VI increased from 0.04 to 0.39, 0.27, 0.24, 0.27, 0.23, and 0.18, respectively (Figure 1). The experimental data indicate that the biodegradability of the dyeing wastewater improved significantly following treatment by the HA-CMBR. Similar to the COD removal performance, at stage V and VI, average BOD_5_/COD decreased by approximately 50%. The results indicate that anaerobic microorganisms were inhibited during stages V and VI.

**Figure 2:**
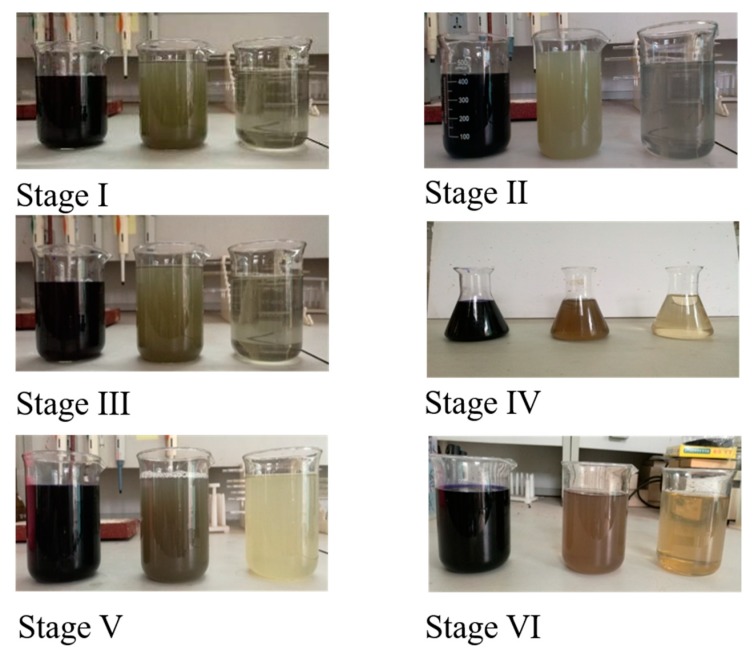
Color changes during the study (left: Influent; middle: Treated by anaerobic microorganisms; right: Filtrate).

Influent chroma and the total chroma removal rate were approximately 1000 and 99%, respectively, in stage I; 2000–2500 and 99.6% in stage II; 2000–2500 and 99.6% in stage III; 3500 and 99.7% in stage IV; 4000 and 99.8% in stage V; and 5000 and 99.5% in stage VI (Figure 2). Chroma removal rates exceeded 99%. During the first four stages, the influent color changed from navy blue to almost white. After filtration through the flat-sheet ceramic membrane, the effluent turned clear, and no obvious color could be identified. Although the chroma removal rates were slightly decreased in stages V and VI, the effluent chroma still met the relevant discharge standards. The results show that the HA-CMBR has good chroma removal performance in treating the dyeing wastewater used in this study. The average TN and TP removal rates were 20% and 6% in the HA-CMBR, which are similar to the results reported by Jin et al. [24]. DO was controlled below 0.5 mg/L. pH was maintained at 7.0–8.0.

### 3.2. Membrane Fouling

During the study, the membrane flux was set at 5 L/m^2^/h. As mentioned previously, the test was divided into six stages. At the end of each phase, the flat-sheet ceramic membranes were chemically cleaned by pumping 1000 mg/L NaOCl into the inner space of the membranes. The membranes were soaked for 1–2 h to recover filtration performance.

**Figure 3:**
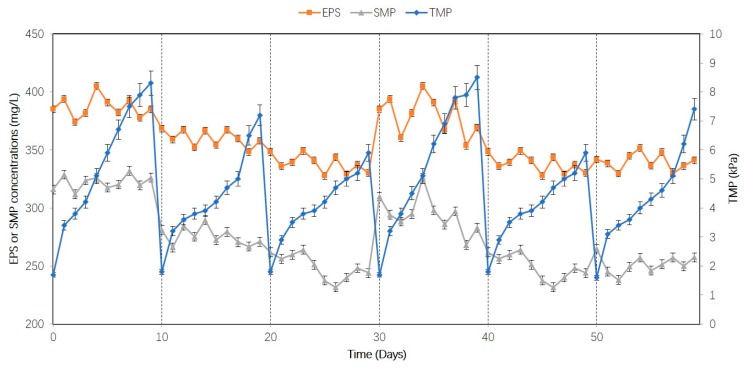
Trans-membrane pressure (TMP), extracellular polymeric substances (EPS), and soluble microbial products (SMP) changes during the study.

Figure 3 shows the TMP, SMP, and EPS changes during the study. TMP increased from 1.7 to 8.3 kPa in stage I (488%); 1.8 to 7.2 kPa in stage II (400%); 1.8 to 5.9 kPa in stage III (328%); 1.7 to 8.5 kPa in stage IV (500%); 1.8 to 5.9 kPa in stage V (328%); and from 1.6 to 7.4 kPa in stage VI (462%). The results show that the TMP of the flat-sheet ceramic membrane was restored to the initial value after chemical cleaning.

EPS and SMP easily adhere to the surface of the membrane, thereby clogging the membrane pores [12,14]. As shown in Figure 3, the EPS and SMP concentrations were closely related to TMP. From stage I to stage III, there were decreasing concentrations of EPS and SMP. Correspondingly, the TMP at the end of each phase decreased from 8.3 kPa to 5.9 kPa. In stage IV, the concentrations of EPS and SMP increased, as did TMP. Thus, TMP increased with decreasing membrane flux. Zhang et al. [12] also found that the decrease in membrane flux was mainly caused by a combination of EPS, SMP, and fine sludge particles attached to the membrane pores and surface. Beyond that, there were subsequent decreases in EPS and SMP concentration during stages V and VI. EPS and SMP were found to be positively related to microbial activity [14]. During stages V and VI, microbial inhibition led to poor COD removal rates and therefore decreasing EPS and SMP. The results show that membrane fouling in this study occurred mainly due to EPS and SMP.

### 3.3. Microbial Diversity

**Figure 4:**
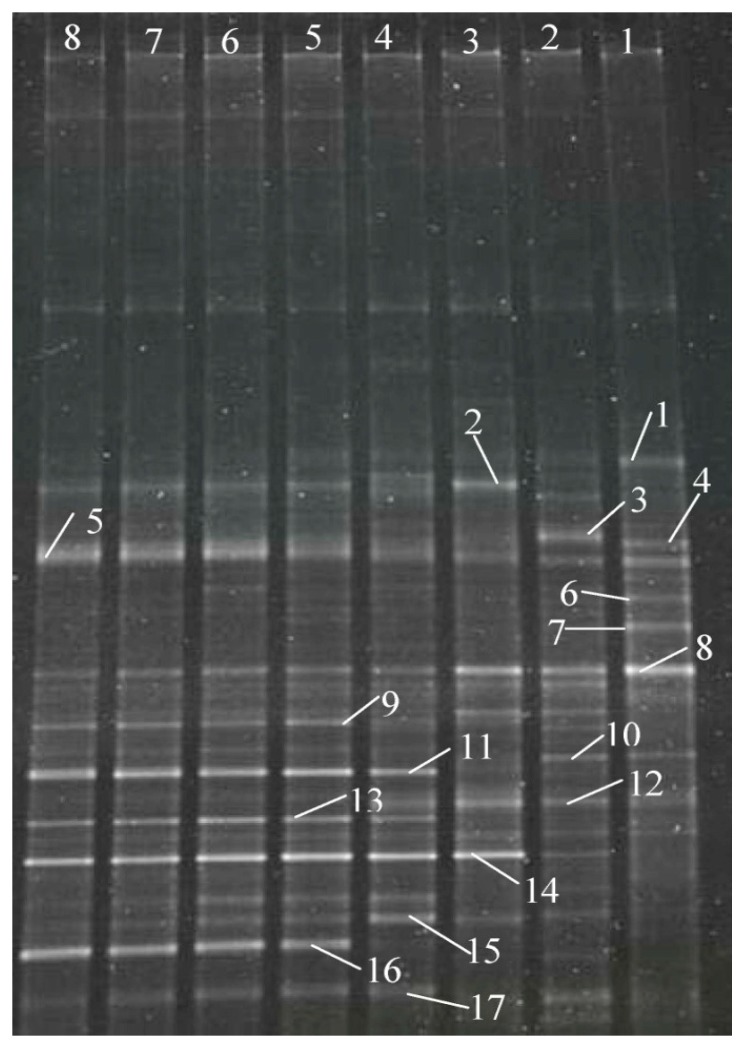
DGGE photos.

At the end of the start-up period, microbial diversity showed an obvious change. The seed sludge showed high microbial diversity, however, after treating the dyeing wastewater (see Figure 4), bands 1, 3, 4, 6, 7, and 10 disappeared, and new bands 2, 12, 14, and 15 dominated in the HA-CMBR. Bands 5, 9, 11, 13, and 16 were further enriched with increasing NLRs (Nos. 4–8), while bands 2, 8, 15, and 17 decreased. The present findings show that bands 5, 9, 11, 13, 14, and 16 can dominate in treating dyeing wastewater.

**Figure 5:**
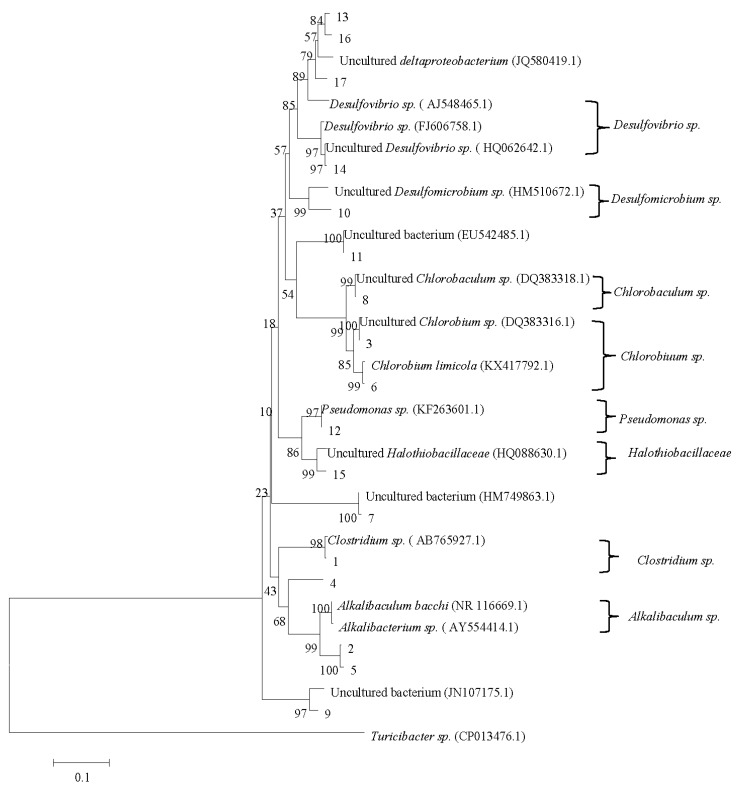
Microbial phylogenetic tree (the ruler length represents 10% divergence. The number of nodes represents confidence).

To evaluate the relationships between the bacteria in the sludge, a microbial phylogenetic tree was built as shown in Figure 5. The identified bands can be clustered into eight genera: *Desulfovibrio* sp., *Desulfomicrobium* sp., *Chlorobaculum* sp., *Chlorobium* sp., *Pseudomonas* sp., *Halothiobacillaceae*, *Clostridium* sp., and *Alkalibaculum* sp. *Desulfovibrio* sp. was reported to have the potential to degrade both Reactive Black 5 and Remazol Brilliant Blue R under hydrolysis acidification conditions, to deal with simulated dyeing wastewater [25]. Li et al. [26] found that *Desulfomicrobium* sp. was abundant in anaerobic sludge used for synergetic decolorization of Reactive Blue 13 effluent.

*Chlorobium* sp. was reported to be dominant in mature biofilm when treating real dyeing wastewater [27]. *Pseudomonas* sp. was identified in a down flow microaerophilic fixed film bioreactor treating reactive azo dyes [28]. *Halothiobacillaceae* was detected in a sequencing batch reactor for cloth printing and dyeing wastewater treatment [29]. *Clostridium* sp. was reported to achieve complete decolorization of Remazol reactive dyes after 24–72 h [30]. *Alkalibaculum* sp. was reported to participate in the degradation of complex organic matter, such as lignocellulosic wastes [31].

**Figure 6:**
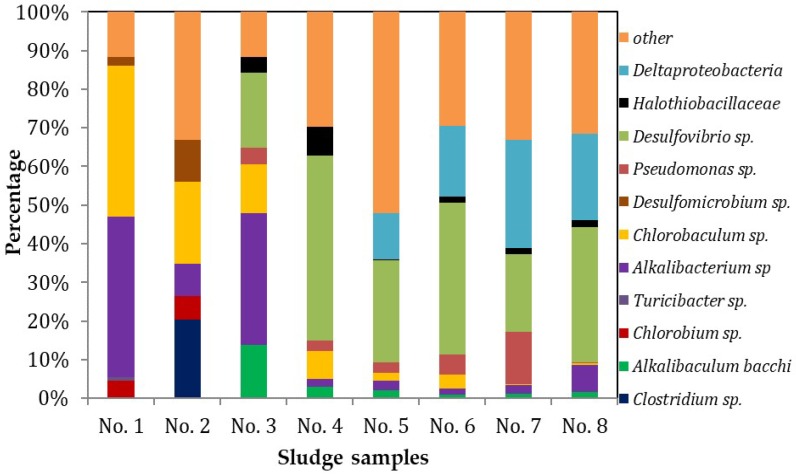
Microbial population relative concentrations during the study.

The relative abundances of bacterial groups (at the phylum level) in the sludge samples are shown in Figure 6. Following the increasing NLRs, *Desulfovibrio* sp., *Deltaproteobacteria*, *Halothiobacillaceae*, *Alkalibaculum* sp., *Pseudomonas* sp., *Desulfomicrobium* sp., and *Chlorobaculum* sp. accounted for approximately 70% of the number of individuals, of which *Desulfovibrio* sp. occupied the largest share. In sludge sample No. 4, the share of *Desulfovibrio* sp. was about 50%. Although the share of *Desulfovibrio* sp. decreased in sludge samples 5 and 6, it still accounted for approximately 30% on average. In sludge samples 5–8, *Deltaproteobacteria* accounted for 20% on average. The results indicate that *Desulfovibrio* sp. and *Deltaproteobacteria* were the most important phyla in the HA-CMBR system used for treating dyeing wastewater in this study. Those phyla with a share of less than 1% are classified as other and together accounted for 30% on average, which means that the sludges were in abundance of biological diversity.

**Figure 7:**
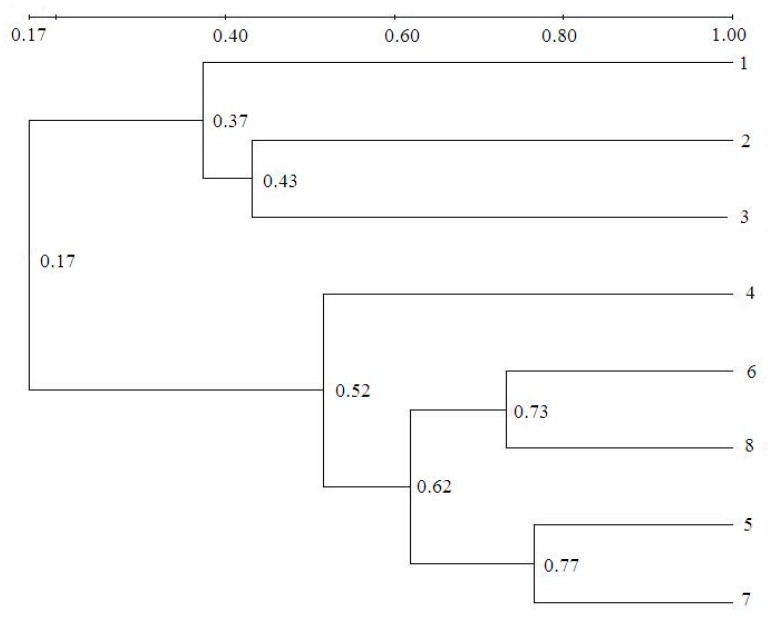
Map clustering tree based on DGGE.

The map clustering tree based on DGGE is shown in Figure 7. In this tree, if a species in a community becomes a species with high similarity in another community, it is considered a small change, and the distance between the two communities is short. On the other hand, if a species in a community becomes another species with a low relationship, there is a large distance between the two communities. There was only 37%–43% similarity between seed sludges and the cultivated sludge sampled during the start-up period, which means that almost 60% of species evolved with the dyeing wastewater in this study. Following the increasing NLRs, the similarity increased. There was 73% similarity between sample Nos. 6 and 8, which further increased to 77% between Nos. 5 and 7. The results indicate that the bacteria in the seed sludge were able to rapidly adapt to the changing feed.

Microbial diversity was analyzed using the Shannon–Weaver index (see Table 2). The diversity indexes of sample Nos. 1 and 2 are 1.412 and 2.325, respectively. Nos. 3–8 have low diversity indexes of 1.935–1.999. No. 1 was collected from a running reactor in the lab, and No. 2 was collected from a real dyeing wastewater treatment plant. The results show that the seed sludge from the real wastewater treatment plant had high microbial diversity. However, following cultivation, microbial diversity decreased, indicating that certain species became dominant at the expense of overall diversity, as shown in Figure 6.

In this study, good chroma removal is deduced to derive from the new strains developed in the HA-CMBR. There are many types of dyes, potentially requiring different types of microorganisms for decolorization. However, in many cases, microbial information is lacking, which is one of the difficulties faced in biodegradation of dyeing wastewater. In this study, microbes that were not contained in the seed gradually emerged and were further enriched after cultivation. This indicates that the bacterial population in the sludge can be changed and used for treating new types of dye wastewater through efficient cultivation. An HA-CMBR can reduce sludge loss by 100%, which is an efficient means of sludge enrichment. Therefore, HA-CMBR in this study is of practical value in treating dyeing wastewater.

## 4. Conclusions

HA-CMBR was used in treating dyeing wastewater. After seven days of operation, the COD removal rate reached approximately 64%, and the chroma removal rate was as high as 99%. COD removal rate decreased sharply when influent COD concentration exceeded 9000 mg/L. The microbial community in the sludge samples showed obvious changes, and the main strains in the HA-CMBR were *Desulfovibrio* sp. (24%), *Deltaproteobacteria* (15%), and *Turicibacter* sp. (37%). The tested AnMBR demonstrates energy-efficient treatment of dye wastewater, and might further enable treatment of different effluent types (through rapidly evolving microbial community structure) and avoidance of biogas emissions/remediation costs.

## Figures and Tables

**Table 1:** Compositions of dying wastewater.

pH	Color	Chroma	Salinity (g/L)	COD (mg/L)	BOD_5_ (mg/L)	TN (mg/L)	NH_4_^+^–N (mg/L)	TP (mg/L)
1.0	Navy blue	10,000	2.5–3	17,000–19,000	680–760	264–300	241–260	0.1–1.7

**Table 2:** Biodiversity change during the study.

No.	Biodiversity
1	1.412
2	2.325
3	1.999
4	1.672
5	1.935
6	1.957
7	1.660
8	1.777
